# Changes in the plasma microvesicle proteome during the ovarian hyperstimulation phase of assisted reproductive technology

**DOI:** 10.1038/s41598-020-70541-w

**Published:** 2020-08-12

**Authors:** Nina Olausson, Fariborz Mobarrez, Roman Zubarev, Alexey Chernobrovkin, Dorothea Rutishauser, Katarina Bremme, Eli Westerlund, Outi Hovatta, Håkan Wallén, Peter Henriksson

**Affiliations:** 1 Department of Clinical Sciences, Danderyd Hospital, Karolinska Institutet, 18288 Stockholm, Sweden; 2 Department of Medical Sciences, Uppsala University, 75185 Uppsala, Sweden; 3 Department of Medical Biochemistry and Biophysics, Karolinska Institutet, 17177 Stockholm, Sweden; 4 Department of Women’s and Children’s Health, Karolinska Institutet, 17177 Stockholm, Sweden; 5 Department of Clinical Science, Intervention and Technology, Karolinska Institutet, 17177 Stockholm, Sweden

**Keywords:** Thromboembolism, Infertility, Proteomics

## Abstract

The incidence of pulmonary and venous thromboembolism is increased during the first trimester of pregnancies after assisted reproductive technology (ART) compared to spontaneous conception. We previously found that haemostatic plasma variables changed but within normal limits during controlled ovarian hyperstimulation (COH) concomitant with a major increase in plasma microvesicles (MVs) and markers indicating cell activation. We now explored the proteome of these MVs. Thirty-one women undergoing ART were blood sampled at down-regulation (DR) of oestrogen and at high level stimulation (HLS) with its 10–100-fold increased oestrogen level. Samples were analysed by liquid chromatography and tandem mass spectrometry to identify and quantify the proteome. We identified 306 proteins in the MVs and 72 had changed significantly at HLS compared to DR and more than 20% of them were associated with haemostasis. Thus, proteins related to both haemostasis and complement activation altered in plasma MVs in parallel with MV activation during COH. This needs to be further explored in the clinical context.

## Introduction

Assisted reproductive technology (ART) is increasingly used to treat infertility since 1978. In vitro fertilisation (IVF) and intracytoplasmic sperm injection (ICSI) are the most applied of the ARTs. ICSI was developed to treat male factor infertility i.e. when the cause was sperm-related. ICSI has today in many clinics replaced IVF irrespective of the cause of infertility. To date more than ten million people have been born after IVF and ICSI^1^.The method is considered effective and safe, with approximately a third of the embryo transfers resulting in a pregnancy^2^. In 2013 we found that the incidence of venous thromboembolism (VTE) increased during the first trimester of ART pregnancies with a sevenfold increase in pulmonary embolism (PE) as compared to matched pregnant women^3^. It is of course of immense importance to understand the pathogenesis of this increased incidence of potentially lethal PE. PE is indeed a dominating cause of maternal mortality^4^. During the controlled ovarian hyperstimulation (COH) phase of ART there is a huge increase in endogenous oestrogen levels. It is thus plausible that oestrogen could have a causal role related to the increased incidence of VTE and PE^5^.

We and others have identified changes in haemostatic variables in plasma during COH. In our studies both thrombin generation and fibrin formation were altered in the direction of a procoagulable state though still within reference values^6^. To assess platelet function in vivo and in vitro is methodologically demanding and there is thus a lack of knowledge of changes in cell-based haemostasis during ART. Microvesicles (MVs) are small vesicles generated and released from cells undergoing cell activation or apoptosis. The cellular origin of MVs can be determined through analysis of membrane bound surface markers with specific monoclonal antibodies. Besides surface markers, MVs can harbour various cytokines, growth factors and proteases connecting them to thrombosis, inflammation and immune responses. We have previously found elevated levels of MVs in patients with acute coronary syndrome^7,8^, type 1 diabetes^9^ and peripheral arterial occlusive disease^10^. Circulating MVs might thus be conceivable carriers of cellular candidate biomarkers that could help to identify high-risk patients or to assess disease activity in patients.

In a previous study we found that circulating cell-derived MVs, with platelet-derived MVs in particular, increased during the COH phase of ART (Table 1)^11^. Besides an increase in total numbers of MVs, we found that MVs with specific markers of activation and inflammation increased significantly. Conceivable mechanistic explanations might be identified by studying changes in the protein composition of MVs during COH. We thus probed modifications of plasma MVs during ART by performing a proteomic analysis of MV-enriched pellets. The protein content of such pellets represents less than 0.003% of the total amount of plasma proteins and changes in this fraction could thus not be detected if plasma was analysed. The plasma MV fraction was dominated by platelet-derived MVs and thus offers a chance to study platelet modification. However, flow cytometric studies are restricted to capture quantitative and qualitative changes of membrane bound proteins, whilst proteomic analysis offers a possibility to capture modifications in the whole proteome of the MVs.

**Table 1:** Baseline characteristics of the 31 patients and previously reported changes in oestradiol level and microvesicles during controlled ovarian hyperstimulation, as presented in our previous study^11^.

	Down-regulation Mean ± SD	High level stimulation Mean ± SD	*P*-value
Age (years)	33.0 ± 3.3		
BMI (kg/m^2^)	24 ± 3.6		
Oestradiol, E2 (pmol/L)	106 ± 31	5,889 ± 4,723	< 0.001

	Median (IQR) (count/µL)	Median (IQR) (count/µL)	
MVs	5,253 (4,095–8,854)	9,565 (4,935–12,671)	< 0.006
PMVs	3,087 (2,464–5,280)	6,681 (3,084–8,475)	< 0.002
NMVs	554 (439–804)	543 (266–620)	< 0.02
MMVs	257 (210–413)	562 (292–689)	< 0.001
EMVs + E-selectin	191 (151–321)	332 (174–439)	< 0.008
EMVs + VE-cadherin	135 (81–190)	135 (81–183)	0.42

*BMI* body mass index, *MVs* microvesicles, *PMVs* platelet microvesicles, *NMVs* neutrophil microvesicles, *MMVs* monocyte microvesicles, *EMVs* endothelial microvesicles, *SD* standard deviation, *IQR* interquartile range.

*P*-value calculated with Wilcoxon signed-rank test.

Our hypothesis is that the increased incidence of PE and deep venous thrombosis (DVT) during the first trimester of pregnancies after ART is initiated by the profound increase in endogenous oestrogen levels during COH. Our approach was thus to explore the cellular response to endogenous oestrogen by assessment of the protein composition of MVs and the qualitative and quantitative changes in their protein constituents during ovarian stimulation. The objective was to enable a better understanding of cellular mechanisms and pathways, both physiological and pathological, affected during ART, which could help to explore potential mechanisms explaining the increased incidence of PE and DVT in ART-pregnancies.

## Results

**Figure 1:**
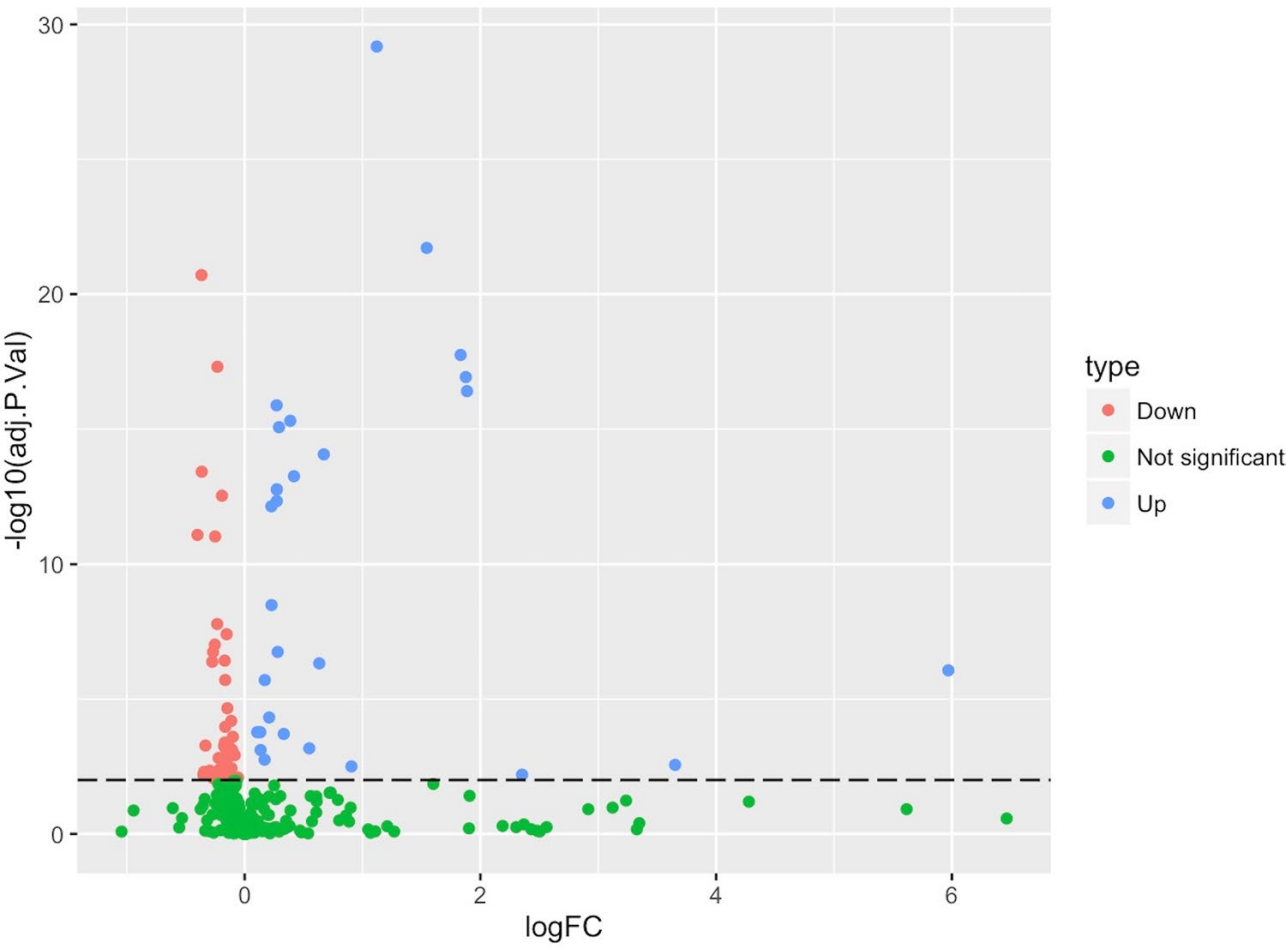
Volcano plot of all proteins (n = 306) and their change during controlled ovarian hyperstimulation. The x-axis represents the logarithm to the base 2 of the fold change (FC) and the y-axis the negative logarithm of the adjusted *P*-value. Dotted line demarks the significance level *P* < 0.01.

We identified 1,199 proteins in the MV fraction of plasma during the COH phase of ART. Out of these identified proteins, 306 were quantified in more than 50% of all samples and were used in further analysis using moderated t-tests for paired comparison of protein abundance at high level stimulation (HLS), with its 10–100-fold increased oestradiol (E2) levels, to the abundance at down-regulation (DR), with E2 levels less than 150 pmol/L^6^. T-test originated *P*-values were corrected for multiple comparisons by the Benjamini–Hochberg method. Proteins with corrected *P*-values less than 0.01 were considered significantly changed and we thus found 72 significantly changed proteins; 28 of those were up-regulated and 44 were down-regulated. (Fig. 1, Table 2, Supplementary Table S1). We found that the dominating cellular compartment of the enriched proteins were vesicle bound with more than half of the up-regulated proteins annotated as blood microvesicle proteins (GO:0072562; Fig. 2)^12^. Furthermore, the most abundant protein in plasma, albumin, was not abundant in any of our samples and these results indicate that the samples constituted a representative MV pellet.

**Table 2:** List of the 72 significantly changed proteins during controlled ovarian hyperstimulation, *P* < 0.01.

Protein name	Gene name	Fold change log_2_	Adjusted *P*-value
**Down-regulated proteins**			
Alpha-1-acid glycoprotein 1	ORM1	− 0.40	< 0.0001
Apolipoprotein E	APOE	− 0.37	< 0.0001
Haptoglobin; Haptoglobin alpha chain; Haptoglobin beta chain	HP	-0.36	< 0.0001
Serum amyloid A-4 protein	SAA2–SAA4; SAA4	− 0.35	0.0063
Ig lambda chain V–V region DEL	NA	− 0.34	0.0050
Coagulation factor V; Coagulation factor V heavy chain; Coagulation factor V light chain	F5	− 0.33	0.0005
Proteoglycan 4; Proteoglycan 4 C-terminal part	PRG4	− 0.30	0.0045
Tetranectin	CLEC3B	− 0.27	< 0.0001
NA	IGKV1–17	− 0.27	< 0.0001
NA	IGLV3–9	− 0.26	0.0087
C4b-binding protein alpha chain	C4BPA	− 0.25	< 0.0001
Plasma protease C1 inhibitor	SERPING1	− 0.25	< 0.0001
Complement component C6	C6	− 0.23	< 0.0001
Antithrombin-III	SERPINC1	− 0.23	< 0.0001
Glutathione peroxidase; Glutathione peroxidase 3	GPX3	− 0.22	0.0015
Carboxylic ester hydrolase; Cholinesterase	BCHE	− 0.21	0.0040
Alpha-1-acid glycoprotein 2	ORM2	− 0.20	0.0069
Vitamin K-dependent protein S	PROS1	− 0.19	< 0.0001
C4b-binding protein beta chain	C4BPB	− 0.19	0.0017
NA	IGLV1–44	− 0.17	0.0006
Extracellular matrix protein 1	ECM1	− 0.17	0.0005
Immunoglobulin J chain	IGJ; JCHAIN	− 0.17	< 0.0001
NA	IGHV3–72	− 0.17	0.0004
Transthyretin	TTR	− 0.17	< 0.0001
Ig lambda-2 chain C regions; Ig lambda-3 chain C regions	IGLC3; IGLC2	− 0.16	0.0001
Apolipoprotein B-100; Apolipoprotein B-48	APOB	− 0.16	0.0009
Gelsolin	GSN	− 0.15	< 0.0001
Complement C1q subcomponent subunit B	C1QB	− 0.15	0.0007
Complement factor I; Complement factor I heavy chain; Complement factor I light chain	CFI	− 0.15	< 0.0001
Apolipoprotein D	APOD	− 0.15	0.0015
Apolipoprotein C-I; Truncated apolipoprotein C-I	APOC1	− 0.14	0.0038
Histidine-rich glycoprotein	HRG	− 0.14	0.0007
Monocyte differentiation antigen CD14; Monocyte differentiation antigen CD14, urinary form; Monocyte differentiation antigen CD14, membrane-bound form	CD14	− 0.13	0.0013
*N*-Acetylmuramoyl-l-alanine amidase	PGLYRP2	− 0.13	0.0006
Apolipoprotein A-II; Proapolipoprotein A-II; Truncated apolipoprotein A-II	APOA2	− 0.12	0.0067
Alpha-2-antiplasmin	SERPINF2	− 0.11	< 0.0001
NA	IGKV3–11	− 0.11	0.0036
Complement C1q subcomponent subunit A	C1QA	− 0.11	0.0097
Complement component C7	C7	− 0.11	0.0008
Ig gamma-1 chain C region	IGHG1	− 0.11	0.0009
Complement component C8 gamma chain	C8G	− 0.10	0.0087
Alpha-1-antichymotrypsin; Alpha-1-antichymotrypsin His-Pro-less	SERPINA3	− 0.10	0.0003
Complement C4-B; Complement C4 beta chain; Complement C4-B alpha chain; C4a anaphylatoxin; C4b-B; C4d-B; Complement C4 gamma chain	C4B	− 0.08	0.0012
Complement factor B; Complement factor B Ba fragment; Complement factor B Bb fragment	CFB	− 0.06	0.0081
**Up-regulated proteins**			
Inter-alpha-trypsin inhibitor heavy chain H4; 70 kDa inter-alpha-trypsin inhibitor heavy chain H4; 35 kDa inter-alpha-trypsin inhibitor heavy chain H4	ITIH4	0.11	0.0002
Apolipoprotein A-I; Proapolipoprotein A-I; Truncated apolipoprotein A-I	APOA1	0.13	0.0002
Kininogen-1; Kininogen-1 heavy chain; T-kinin; Bradykinin; Lysyl-bradykinin; Kininogen-1 light chain; Low molecular weight growth-promoting factor	KNG1	0.14	0.0008
Fibulin-1	FBLN1	0.17	0.0018
Fibrinogen gamma chain	FGG	0.17	< 0.0001
Alpha-2-HS-glycoprotein; Alpha-2-HS-glycoprotein chain A; Alpha-2-HS-glycoprotein chain B	AHSG	0.21	< 0.0001
Fibrinogen alpha chain; Fibrinopeptide A; Fibrinogen alpha chain	FGA	0.23	< 0.0001
Fibrinogen beta chain; Fibrinopeptide B; Fibrinogen beta chain	FGB	0.23	< 0.0001
Alpha-1-antitrypsin; Short peptide from AAT	SERPINA1	0.27	< 0.0001
Thyroxine-binding globulin	SERPINA7	0.27	< 0.0001
Vitamin D-binding protein	GC	0.27	< 0.0001
Hyaluronan-binding protein 2; Hyaluronan-binding protein 2 50 kDa heavy chain;Hyaluronan-binding protein 2 50 kDa heavy chain alternate form; Hyaluronan-binding protein 2 27 kDa light chain; Hyaluronan-binding protein 2 27 kDa light chain alternate form	HABP2	0.28	< 0.0001
Ceruloplasmin	CP	0.29	< 0.0001
von Willebrand factor; von Willebrand antigen 2	VWF	0.33	0.0002
Vitronectin; Vitronectin V65 subunit; Vitronectin V10 subunit; Somatomedin-B	VTN	0.39	< 0.0001
Corticosteroid-binding globulin	SERPINA6	0.42	< 0.0001
NA	NA	0.55	0.0007
Lipopolysaccharide-binding protein	LBP	0.63	< 0.0001
Fetuin-B	FETUB	0.67	< 0.0001
Actin, cytoplasmic 1;Actin, cytoplasmic 1, N-terminally processed	ACTB	0.91	0.0031
Angiotensinogen;Angiotensin-1;Angiotensin-2;Angiotensin-3;Angiotensin-4;Angiotensin 1–9;Angiotensin 1–7;Angiotensin 1–5;Angiotensin 1–4	AGT	1.12	< 0.0001
Sex hormone-binding globulin	SHBG	1.54	< 0.0001
Pregnancy zone protein	PZP	1.83	< 0.0001
Hemoglobin subunit beta;LVV-hemorphin-7;Spinorphin	HBB	1.88	< 0.0001
Hemoglobin subunit alpha	HBA1	1.89	< 0.0001
Heat shock cognate 71 kDa protein	HSPA8	2.35	0.0063
78 kDa glucose-regulated protein	HSPA5	3.65	0.0027
Carbamoyl-phosphate synthase [ammonia], mitochondrial	CPS1	5.97	< 0.0001

*Log*_*2*_ logarithm to the base 2, *NA* not applicable.

**Figure 2:**
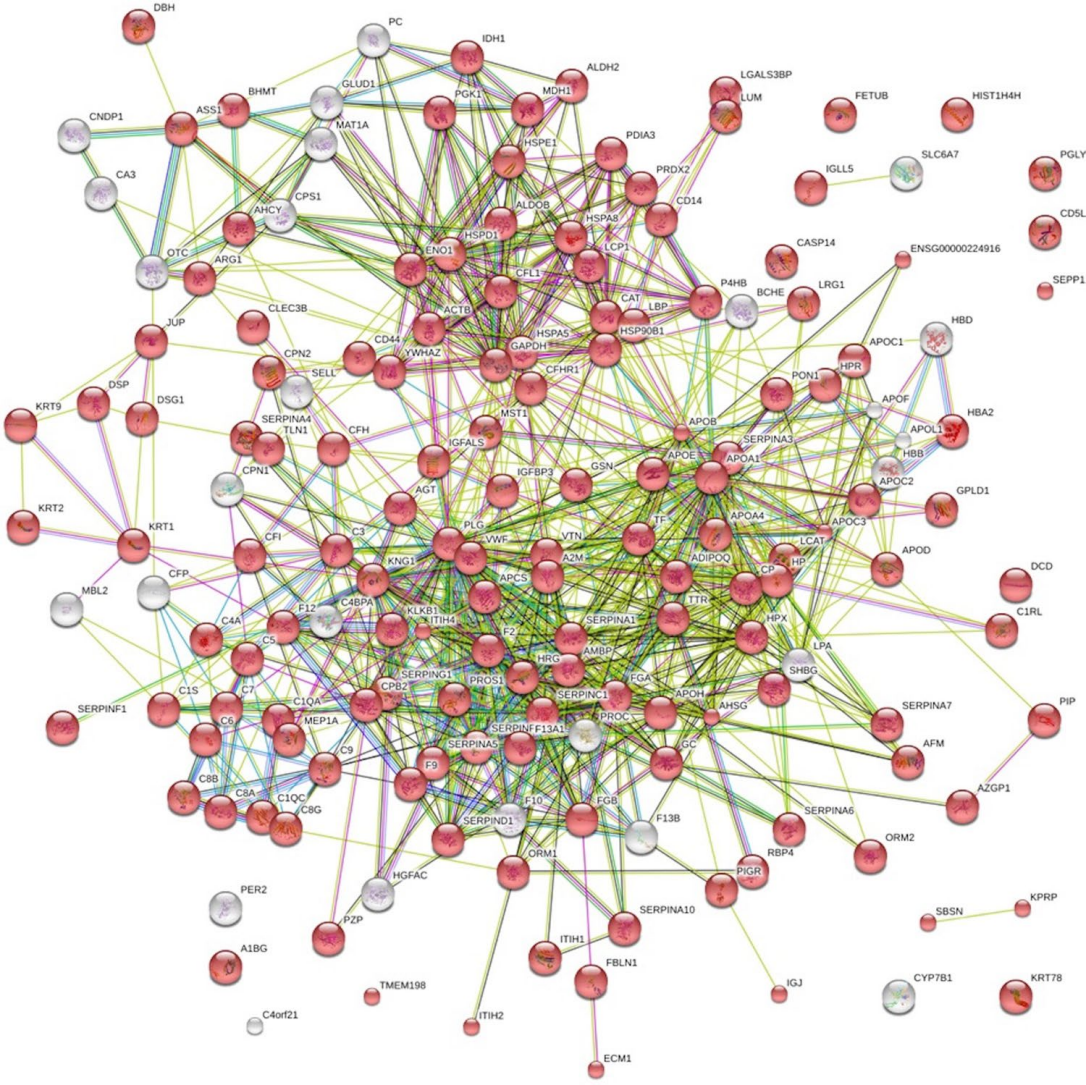
Protein-to-protein interaction showing in red colour the proteins identified as blood microvesicle-bound (n = 162 of 206 recognized). The network diagram was originally obtained with STRING database^12^.

**Figure 3:**
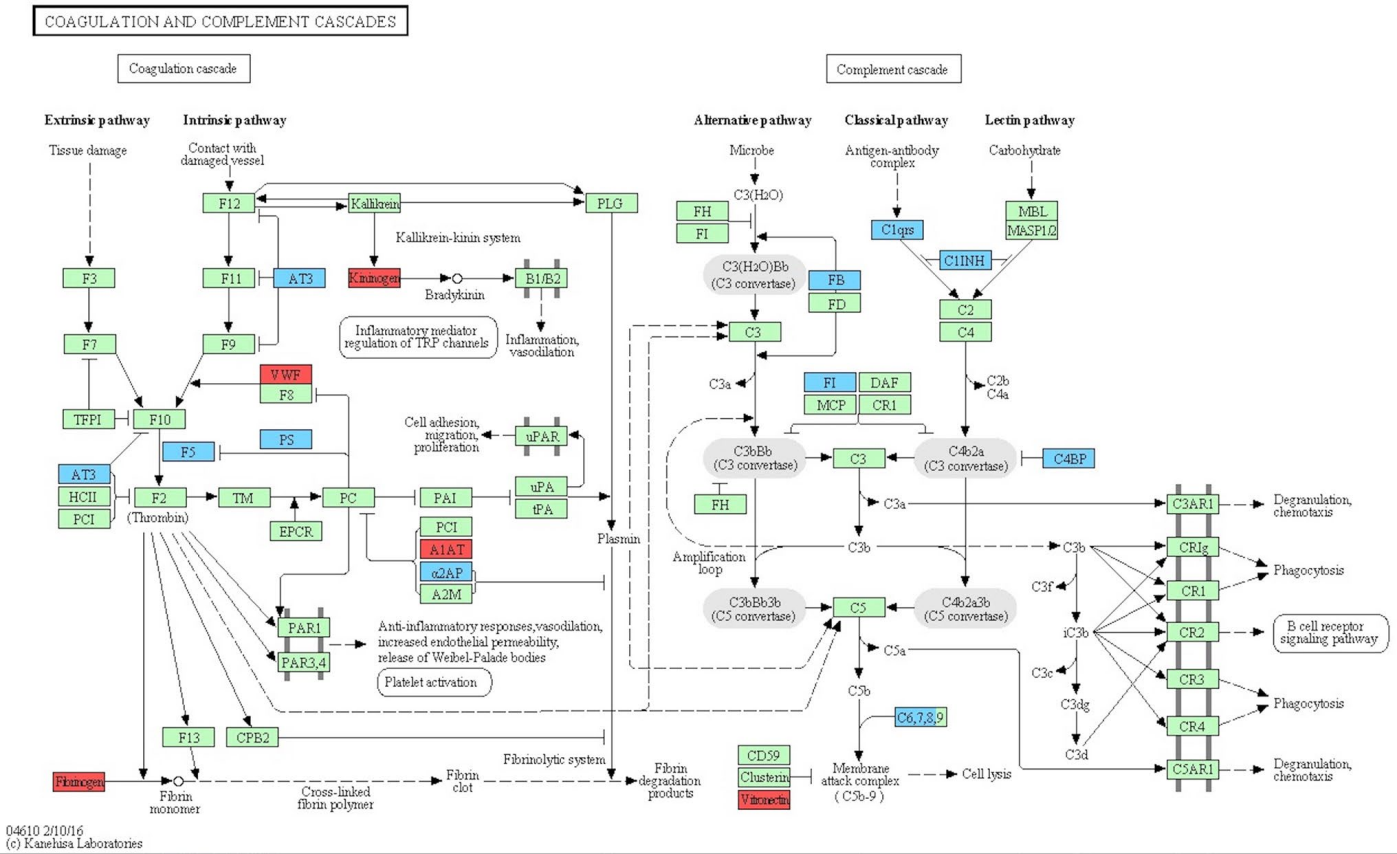
Coagulation and complement cascade proteins significantly altered (*P* < 0.01) during controlled ovarian hyperstimulation (n = 18). Red denotes up-regulated proteins. Blue denotes down-regulated proteins. The diagram was originally obtained from the KEGG database^13^.

A large number of the altered proteins are associated to the coagulation cascade but also to the complement cascade. Most of these proteins can be seen in the picture using the KEGG database (Fig. 3)^13^. Dominating pathways and most enriched biological processes were those of blood coagulation and fibrinolysis, including more than a third of the identified pathways.

## Discussion

We found significant alterations in the MV proteome related to coagulation. The potential implications of these findings and other identified proteins that might be of relevance will be discussed.

We detected several shifts in protein levels that might be anticipated following the increased oestrogen level during COH. One example is the up-regulation of oestrogen sensitive Sex Hormone Binding Globulin (SHBG), which is the main carrier of E2 in the circulation. This has previously been found in plasma during COH^14^. Furthermore, the acute phase protein alpha-1-antitrypsin (SERPINA1) was up-regulated and has previously been found to increase in pregnancy and to decrease in post-menopausal women^15^. It has been speculated that this protein could be related to a successful pregnancy^16^. Moreover, oestrogen might trigger secretion of von Willebrand Factor (VWF) from endothelial cells and in line with this we found VWF to be up-regulated in MVs at HLS. Other MV proteins decreased concurrently with the increase in oestrogen levels. We found decreased antithrombin, which has previously been found to decrease in plasma following the increased oestrogen level during COH^17^.

Furthermore, we found alterations in coagulation proteins, as mentioned and illustrated in Fig. 3, with an up-regulation in MVs of VWF and fibrinogen, which are both essential for clot formation in the conversion of fibrinogen to fibrin when the coagulation system is activated. VWF is a large multimeric, adhesive protein important to normal haemostasis adhering both to the subendothelium, the site of injury, and to platelet-surface-receptors. VWF in plasma increases in response to inflammation and is released from the endothelium as well as from platelets upon activation.

We found that kininogen-1 (KNG1), vitronectin (VTN) and alpha-1-antitrypsin (SERPINA1) were up-regulated, while the natural anticoagulants, antithrombin (SERPINC1) and protein S (PROS1), were down-regulated. High-molecular-weight-kininogen can be expressed by activated platelets on their surface membrane and the protein is crucial to the intrinsic system of coagulation as a co-factor for prekallikrein, FIX and FXII, positioning them for facilitation of activation. VTN has been shown to be incorporated in the fibrin clot^18^. It is a cofactor of plasminogen activator inhibitor (PAI-1), binds to and stabilises PAI-1, one of the most important inhibitors of fibrinolysis, through inhibition of plasminogen activator (tPA). Thus, increased levels of VTN might decrease fibrinolysis. Furthermore, VTN has been shown to augment platelet adhesion and platelet aggregation in the formation a blood clot^19^.

Apart from the proteins shown in Fig. 3, there were other proteins with substantial alterations that might affect haemostasis, such as histidine-rich glycoprotein (HRG) and tetranectin (CLEC3B). These were found to be down-regulated. HRG circulates in plasma and is found in the alpha-granules of platelets which are released during platelet activation. HRG binds to FXIIa and inhibits contact activation and can also bind to fibrinogen and plasminogen and thus inhibit both coagulation and fibrinolysis^20^. Animal studies have shown that HRG-deficient mice have an accelerated arterial thrombus formation^21^. These findings suggest that HRG may act both as an anticoagulant and as an antifibrinolytic modifier and might thus control platelet function in vivo. CLEC3B binds to the kringle 4 region of plasminogen and activates the change of plasminogen to plasmin and might thus have a potential to interact and decrease fibrinolysis. Decreased levels have been shown in coronary artery disease^22^.

We also found nine significantly down-regulated proteins associated to the complement system during COH (*P* < 0.01). The complement system is in turn closely connected to the similarly structured coagulation system with an interaction during inflammation, healing of tissue damage and in the development of a thrombosis. We found down-regulation of C1-inhibitor (C1INH), which acts by binding to FXII and kallikrein and thus inhibits activation of the intrinsic system. It further inhibits thrombin directly and plasmin. C1INH inhibits the first component of the complement system, i.e. complement C1r and C1s. Decreased levels of complement factors have been found in human follicular fluid during ART and complement factor I has been suggested to be a biomarker for fertilisation^23^.

Fetuin-B (FETUB) was also found to increase. FETUB is a member of the Cystatin superfamily and is increased in females. This protein is required for egg fertilisation and loss of FETUB decreases fertility due to hardening of the Zona pellucida membrane that surrounds the oocyte, thereby hindering binding and penetration of the sperm. A recent study indicated an association of high FETUB concentrations in serum with fertilisation rate in ART^24^. FETUB has not been extensively studied but has been found to increase in patients with acute myocardial infarction^25^ and in other states of increased cardiovascular risk such as in females treated by oestrogen in Women´s Health Initiative^26^. FETUB is thus both related to fertility and cardiovascular risk and should indeed be further studied.

In addition, we also detected further alterations in potential risk markers of thrombosis. Fibulin-1 (FBLN1) was up-regulated in MVs. This is a protein linked to thrombosis by its binding to fibrinogen, mediating platelet adhesion^27^, and has been proposed to be a biomarker for cardiovascular disease in patients with diabetes and kidney disease^28^.

As described above we found changes in MVs of several proteins previously known to change during COH, but also some sparsely detected protein changes. A link between the supraphysiologic oestrogen levels during COH and a procoagulant and proinflammatory state was strengthened by the present results identified in MVs.

With regard to the limitations of our study, it is worth noting that the population of study was small. We detected MV proteome alterations with statistical significance since we compared samples before and after COH with each woman being her own control. The changes found might though be of clinical relevance and help to reveal mechanisms explaining the increased incidence of VTE and PE in ART-pregnancies and open up for further studies. VTE is a rare event afflicting about one to two out of 1,000 pregnant women. Despite the major relative increase during the first trimester—sevenfold as concerns PE—in ART-pregnancies^3^ any associations between thromboembolic events and changes in the MV proteome should require a rather large study.

We analysed MV-enriched samples with the majority of those being platelet-derived MVs, but in the present study we could not derive these proteins to a specific parent cell. The whole proteome and alterations of the proteome before and after COH were analysed, thus including both membrane-bound and proteins found within the MV. Furthermore, our technique has a limit in the detection of low abundance proteins.

## Conclusion

Haemostatic pathway proteins were identified and shown to alter in plasma MVs during the controlled ovarian hyperstimulation phase of ART. These changes thus occurred in the phase of the ART procedure that directly precedes the first trimester with its increased incidence of PE and VTE. It is of utmost importance to elucidate the potential mechanisms behind this serious side effect of ART. Such findings might be potential biomarkers that could be used to identify women at risk of VTE and might indicate a need of adequate prophylactic treatment. Identification of biomarkers might lead to early detection of increased risk and offer a possibility to prevent VTE and potentially lethal PE. Identification of the pathophysiological mechanisms might in the future lead to effective treatment and prophylaxis.

Some of the proteome alterations found might also be useful as potential biomarkers of successful ART; reflecting the processes of oocyte maturation, fertilisation, embryogenesis and implantation, and other alterations might be biomarkers reflecting detrimental processes during ART such as those causing a procoagulant, prothrombotic state.

The clinical usefulness of identified changes in protein levels will necessitate longitudinal studies with large patient cohorts and thus enough cardiovascular events to investigate their potential in the prediction and prevention of events such as PE and VTE in women undergoing ART.

## Methods

### Study population

The present study population and sampling has previously been described. It comprised thirty-one women aged 25–38 years who were treated by ART at the Fertility Unit of Karolinska University Hospital, Stockholm (Table 1)^6^. The study was approved by the Regional Ethical Review Board in Stockholm, reference number 2006/1223-31/1. All patients gave their informed consent to participate. The research was conducted in accordance with the Declaration of Helsinki on ethical principles in medical research. Women with a family history of arterial or venous thrombosis, polycystic ovarian syndrome or ongoing anticoagulation were excluded and no woman was afflicted by arterial hypertension, diabetes mellitus or dyslipidaemia. As described the power exceeded 80% to detect a 20% difference at a two-sided alpha level of 0.05^6^.

As previously described, the long protocol for the ovarian stimulation phase of ART was applied^6^. This was initiated by the gonadotropin releasing hormone (GnRH) agonist, Buserelin (Suprecur, Aventis Pharma, Frankfurt, Germany), to down-regulate oestrogen synthesis. GnRH was given in the form of a nasal spray, 300 µg three times daily, starting on the 21st day of the menstrual cycle. E2 levels were assessed after the menstrual bleeding two weeks later. Ovarian stimulation started when E2 was less than 150 pmol/L. Recombinant follicular stimulating hormone (FSH) was initiated as daily subcutaneous injections of 75–300 IU daily of follitropin alpha, Gonal-f (Merck Serono, Stockholm, Sweden) or follitropin beta, Puregon (Schering-Plough, Gothenburg, Sweden). Concomitantly, the GnRH-agonist was reduced to twice daily. E2 was assessed after 6 days and an ultrasound of the ovaries was performed at day 9–10 after initiation of FSH as previously described^6^.

The women were fasting when they left blood samples, first at the time point maximal down-regulation (DR) and the second time at high level stimulation (HLS), immediately before they received human chorionic gonadotropin (hCG). Samples were taken by direct venepuncture into a medium of buffered sodium citrate (nine parts blood and one part of sodium citrate, 0.129 mol/L; pH 7.4). The samples were centrifuged at 2000g for 20 min at room temperature (RT) to obtain platelet-poor plasma and plasma aliquots were stored at the temperature of − 80 °C.

### Microvesicle enrichment

To prepare a MV-enrich pellet for proteomic analysis, a series of centrifugation steps were performed, as described elsewhere^29^. Briefly, the stored PPP (500 µl) was thawed at 37 °C for approximately 5 min. After thawing, the plasma was centrifuged at 2000g for 20 min at RT to exclude any large particles or cells that might interfere with the analysis. The supernatant was then centrifuged at 20,800g for 45 min at RT. The upper part of the supernatant was discarded (450 µL) and the remaining 50 µL pellet was mixed with 450 µl of PBS (Phosphate-buffered saline, pH 7.4) and centrifuged again at 20,800g for 45 min in RT. After centrifugation, the upper part of the supernatant was discarded (450 µL) and the remaining 50 µl of MV-enriched pellet was vortexed for 10–15 s. The prepared MV-samples was then stored in − 80 °C until proteomics analysis.

### Microvesicle proteomics: protein digestion

The MV enriched plasma samples were thawed on ice and homogenised by vortexing. Small aliquots were mixed 1:8 with 0.5 M Urea and 2.5% methanol and the protein concentration was determined using Micro BCA from Pierce (Thermo Fisher Scientific Inc). From each sample 2 × 10 µg of protein was dissolved in a final concentration of 0.1% ProteaseMax (Thermo Fisher Scientific Inc), 0.05 M Urea, 50 mM ammonium bicarbonate and 10% methanol in a total volume of 80 µL. The resulting protein solutions were sonicated (2 cycles, each cycle 20 s with 40% amplitude) on ice using a probe (Vibra-Cell CV18, Sonics & Materials, Newtown, CT, USA) followed by incubation for 30 min at 37 °C while shaking. Samples were centrifuged and directly subjected to a tryptic digestion protocol carried out by a liquid handling robot (MultiProbe II, Perkin Elmer) as previously described^30^. This included protein reduction in 5 mM DTT at 56 °C and alkylation in 15 mM iodacetamide for 30 min at RT in the dark. Trypsin was added in an enzyme to protein ratio of 1:30 and digestion was carried out over night at 37 °C. Samples were acidified by adding 6 µL concentrated formic acid, incubated for 30 min at RT and centrifuged for 20 min at 1,000g to remove undigested material.

### Microvesicle proteomics: liquid chromatography tandem mass spectrometry

Proteomic analysis was initiated with a pilot study of four samples, of which one sample was run in two replicates. The results were consistent with previous findings in the research group, that the biological variation between individuals was larger than the technical variation between samples due to handling of the samples and technique.

As previously described, tryptic peptides were cleaned and injected into an UltiMate Nano system (Thermo Scientific, Bremen, Germany) in-line coupled to a Q Exactive mass spectrometer (Thermo Scientific, Bremen, Germany)^30^. The chromatographic separation of the peptides was achieved using a 25 cm long, ID 150 µm in-house packed column (C18-AQ ReproSil-Pur, Dr. Maisch GmbH, Germany) at 55 °C with the following gradient: 4 − 30% acetonitrile in 89 min, 30 − 95% ACN for 5 min and 95% ACN for 8 min all at a flow rate of 250 nL/min, as previously described^30^.

As also previously described, the MS-acquisition method comprised of one survey full scan ranging from m/z 300 to m/z 1,650 acquired with a resolution of R = 140,000 at m/z 200 and with a target value of 5e6, followed by data-dependent higher-energy collisional dissociation fragmentation scans from maximum sixteen most intense precursor ions with a charge state greater than or equal to 2^30^. Sequencing was accomplished with a target value of 2e5 ions as determined by predictive automatic gain control, for which the isolation of precursors was performed with a window of 4 m/z. Scans were acquired as profile data with a resolution of R = 17,500 and normalised collision energy was set to 26.

### Data analysis

MaxQuant software (version 1.5.3.30) was used for analysis of MS raw data^31^. We used a false discovery rate (FDR) of 0.01 for proteins and peptides and required a minimum peptide length of six amino acids as described in a previous study^32^. We improved the mass accuracy of precursor ions by the MaxQuant time-dependent recalibration algorithm and as described in the previously mentioned study^32^ we used the Andromeda search engine to search MS/MS spectra against the Uniprot human database combined with 262 common contaminants and concatenated with reversed versions of all sequences. Enzyme specificity was set to trypsin specificity and further modifications were cysteine carbamidomethylation (fixed) as well as protein N-terminal acetylation, asparagine and glutamine deamidation and methionine oxidation (variable) as previously described^32^. MaxQuant was used for identification of the peptides and a maximum of two missed cleavages was allowed. Peptide identification was based on a search with an initial mass deviation of the precursor ion of up to 7 ppm. The fragment mass tolerance was set to 20 ppm. Only proteins quantified with at least two peptides were considered for quantitation. As shown by Lyutvinskiy et al. accurate relative quantification does not require internal standard^33^.

Normalisation of the data was performed by calculating the summed intensities of all proteins in each sample and the median of all these summed intensities over the entire sample set. Each quantitative value was multiplied by the median/summed intensity and the resulting values were log2-transformed. Differences in relative protein abundances between DR and HLS were assessed by moderated paired t-test using limma package^34^. Benjamini–Hochberg corrections for multiple comparisons were used. Gene ontology enrichment analyses was performed in software databases; DAVID^35^, STRING^12^, PANTHER^36^ and KEGG^13^.

## Supplementary information

Supplementary Tables.

## Data Availability

The datasets analysed during the current study are available from the corresponding author on reasonable request.
