# Supplementary material for: Changes in the plasma microvesicle proteome during the ovarian hyperstimulation phase of assisted reproductive technology
Source: Sci Rep. 2020 Aug 12;10:13645. doi: 10.1038/s41598-020-70541-w (PMC7423945; doi:10.1038/s41598-020-70541-w)
Supplement: Supplementary file 1 — Supplementary Tables. [file 41598_2020_70541_MOESM1_ESM.pdf]

SUPPLEMENTARY INFORMATION

Changes in the plasma microvesicle proteome during the ovarian hyperstimulation phase of assisted reproductive technology

Nina Olausson[1], Fariborz Mobarrez[2], Roman Zubarev[3], Alexey Chernobrovkin[3], Dorothea Rutishauser[3], Katarina Bremme[4], Eli Westerlund[1], Outi Hovatta[5], Håkan Wallén[1], Peter Henriksson[1].

[1]Karolinska Institutet, Department of Clinical Sciences, Danderyd Hospital, Karolinska Institutet, 18288 Stockholm, Sweden; [2]Uppsala University, Department of Medical Sciences, 75185 Uppsala, Sweden; [3] Karolinska Institutet, Department of Medical Biochemistry and Biophysics, 17177 Stockholm, Sweden; [4] Karolinska Institutet, Department of Women's and Children's Health, 17177 Stockholm. [5] Karolinska Institutet, Department of Clinical Science, Intervention and Technology, 17177 Stockholm, Sweden.

Supplementary table S1

306 proteins identified in microvesicles during controlled ovarian hyperstimulation.

Protein name	Protein ID
Alpha-1-acid glycoprotein 1	ORM1
Apolipoprotein E	APOE
Haptoglobin; Haptoglobin alpha chain;Haptoglobin beta chain	HP
Serum amyloid A-4 protein	SAA2-SAA4;SAA4
Ig lambda chain V-V region DEL	NA
Coagulation factor V;Coagulation factor V heavy chain;Coagulation factor V light chain	F5
Proteoglycan 4;Proteoglycan 4 C-terminal part	PRG4
Tetranectin	CLEC3B
NA	IGKV1-17
NA	IGLV3-9
C4b-binding protein alpha chain	C4BPA
Plasma protease C1 inhibitor	SERPING1
Complement component C6	C6
Antithrombin-III	SERPINC1
Glutathione peroxidase;Glutathione peroxidase 3	GPX3

Carboxylic ester hydrolase;Cholinesterase	BCHE
Alpha-1-acid glycoprotein 2	ORM2
Vitamin K-dependent protein S	PROS1
C4b-binding protein beta chain	C4BPB
NA	IGLV1-44
Extracellular matrix protein 1	ECM1
Immunoglobulin J chain	IGJ;JCHAIN
NA	IGHV3-72
Transthyretin	TTR
Ig lambda-2 chain C regions;Ig lambda-3 chain C regions	IGLC3;IGLC2
Apolipoprotein B-100;Apolipoprotein B-48	APOB
Gelsolin	GSN
Complement C1q subcomponent subunit B	C1QB
Complement factor I;Complement factor I heavy chain;Complement factor I light chain	CFI
Apolipoprotein D	APOD
Apolipoprotein C-I;Truncated apolipoprotein C-I	APOC1
Histidine-rich glycoprotein	HRG
Monocyte differentiation antigen CD14;Monocyte differentiation antigen CD14, urinary form;Monocyte differentiation antigen CD14, membrane-bound form	CD14
N-acetylmuramoyl-L-alanine amidase	PGLYRP2
Apolipoprotein A-II;Proapolipoprotein A-II;Truncated apolipoprotein A-II	APOA2
Alpha-2-antiplasmin	SERPINF2
NA	IGKV3-11
Complement C1q subcomponent subunit A	C1QA
Complement component C7	C7
Ig gamma-1 chain C region	IGHG1
Complement component C8 gamma chain	C8G

Alpha-1-antichymotrypsin;Alpha-1-antichymotrypsin His-Pro-less	SERPINA3
Complement C4-B;Complement C4 beta chain;Complement C4-B alpha chain;C4a anaphylatoxin;C4b-B;C4d-B;Complement C4 gamma chain	C4B
Complement factor B;Complement factor B Ba fragment;Complement factor B Bb fragment	CFB
Inter-alpha-trypsin inhibitor heavy chain H4;70 kDa inter-alpha-trypsin inhibitor heavy chain H4;35 kDa inter-alpha-trypsin inhibitor heavy chain H4	ITIH4
Apolipoprotein A-I;Proapolipoprotein A-I;Truncated apolipoprotein A-I	APOA1
Kininogen-1;Kininogen-1 heavy chain;T-kinin;Bradykinin;Lysyl-bradykinin;Kininogen-1 light chain;Low molecular weight growth-promoting factor	KNG1
Fibulin-1	FBLN1
Fibrinogen gamma chain	FGG
Alpha-2-HS-glycoprotein;Alpha-2-HS-glycoprotein chain A;Alpha-2-HS-glycoprotein chain B	AHSG
Fibrinogen alpha chain;Fibrinopeptide A;Fibrinogen alpha chain	FGA
Fibrinogen beta chain;Fibrinopeptide B;Fibrinogen beta chain	FGB
Alpha-1-antitrypsin;Short peptide from AAT	SERPINA1
Thyroxine-binding globulin	SERPINA7
Vitamin D-binding protein	GC
Hyaluronan-binding protein 2;Hyaluronan-binding protein 2 50 kDa heavy chain;Hyaluronan-binding protein 2 50 kDa heavy chain alternate form;Hyaluronan-binding protein 2 27 kDa light chain;Hyaluronan-binding protein 2 27 kDa light chain alternate form	HABP2
Ceruloplasmin	CP
von Willebrand factor;von Willebrand antigen 2	VWF
Vitronectin;Vitronectin V65 subunit;Vitronectin V10 subunit;Somatomedin-B	VTN
Corticosteroid-binding globulin	SERPINA6
NA	NA

Lipopolysaccharide-binding protein	LBP
Fetuin-B	FETUB
Actin, cytoplasmic 1;Actin, cytoplasmic 1, N-terminally processed	ACTB
Angiotensinogen;Angiotensin-1;Angiotensin-2;Angiotensin-3;Angiotensin-4;Angiotensin 1-9;Angiotensin 1-7;Angiotensin 1-5;Angiotensin 1-4	AGT
Sex hormone-binding globulin	SHBG
Pregnancy zone protein	PZP
Hemoglobin subunit beta;LVV-hemorphin-7;Spinorphin	HBB
Hemoglobin subunit alpha	HBA1
Heat shock cognate 71 kDa protein	HSPA8
78 kDa glucose-regulated protein	HSPA5
Carbamoyl-phosphate synthase [ammonia]go, mitochondrial	CPS1
Protein AMBP;Alpha-1-microglobulin;Inter-alpha-trypsin inhibitor light chain;Trypstatin	AMBP
Immunoglobulin lambda-like polypeptide 5;Ig lambda-1 chain C regions	IGLL5;IGLC1
Hemoglobin subunit delta	HBD
Ig kappa chain V-I region Walker	NA
Ig kappa chain V-II region MIL	NA
Complement component C8 alpha chain	C8A
Clusterin;Clusterin beta chain;Clusterin alpha chain;Clusterin	CLU
NA	IGKV1D-33
Ig lambda chain V-I region NIG-64;Ig lambda chain V-I region BL2	IGLV1-51
Pigment epithelium-derived factor	SERPINF1
Complement component C8 beta chain	C8B
Ig alpha-2 chain C region	IGHA2
Ig lambda chain V-I region NEW	NA
Fructose-bisphosphate aldolase B;Fructose-bisphosphate aldolase	ALDOB
NA	GC

Properdin	CFP
Carboxypeptidase N subunit 2	CPN2
Alpha-enolase	ENO1
Ig kappa chain V-I region DEE	NA
Mannose-binding protein C	MBL2
Keratin, type II cytoskeletal 2 epidermal	KRT2
Beta-2-microglobulin;Beta-2-microglobulin;Beta-2-microglobulin form pI 5.3	B2M
Keratin, type I cytoskeletal 9	KRT9
Argininosuccinate synthase	ASS1
Alpha-1B-glycoprotein	A1BG
Ig lambda chain V-IV region MOL	NA
Ig heavy chain V-III region GAL	NA
Dermcidin;Survival-promoting peptide;DCD-1	DCD
NA	IGLV8-61
Ig lambda chain V region 4A	IGLV7-43
NA	IGHV3-30
Keratin, type II cytoskeletal 1	KRT1
Peroxiredoxin-1	PRDX1
NA	IGKV1-5
Phosphatidylcholine-sterol acyltransferase	LCAT
NA	IGHV2-26
IgGFc-binding protein	FCGBP
Peroxiredoxin-2	PRDX2
Keratin, type I cytoskeletal 10	KRT10
Complement factor H-related protein 1	CFHR1
Ig mu chain C region;Ig mu heavy chain disease protein	IGHM
Glutathione S-transferase;Glutathione S-transferase A5	GSTA5;GSTA3
Fibulin-1	FBLN1

Serum amyloid P-component;Serum amyloid P-component(1-203)	APCS
Complement factor H	CFH
Tubulin alpha-4A chain	TUBA4A
Keratin, type I cytoskeletal 14	KRT14
Hepatocyte growth factor activator;Hepatocyte growth factor activator short chain;Hepatocyte growth factor activator long chain	HGFAC
Filamin-A	FLNA
Coagulation factor XII;Coagulation factor XIIa heavy chain;Beta-factor XIIa part 1;Coagulation factor XIIa light chain	F12
Beta-Ala-His dipeptidase	CNDP1
Ig alpha-1 chain C region	IGHA1
Leucine-rich alpha-2-glycoprotein	LRG1
Protein ZGRF1	ZGRF1
Beta-2-glycoprotein 1	APOH
Inter-alpha-trypsin inhibitor heavy chain H1	ITIH1
NA	IGKV6-21
Ig heavy chain V-III region BUT	NA
Carboxypeptidase B2	CPB2
Ig heavy chain V-III region TIL;Ig heavy chain V-III region POM	NA
NA	IGKV2-24;IGKV2D-24
Ig heavy chain V-II region SESS	NA
Ig lambda chain V-I region WAH	NA
10 kDa heat shock protein, mitochondrial	HSPE1;HSPE1-MOB4
CD5 antigen-like	CD5L
Malate dehydrogenase, cytoplasmic;Malate dehydrogenase	MDH1
Glutamate dehydrogenase 1, mitochondrial;Glutamate dehydrogenase 2, mitochondrial	GLUD1;GLUD2
Ig heavy chain V-II region WAH	NA

Phosphoglycerate kinase 1	PGK1
Ig kappa chain V-III region NG9	NA
Complement C3;Complement C3 beta chain;C3-beta-c;Complement C3 alpha chain;C3a anaphylatoxin;Acylation stimulating protein;Complement C3b alpha chain;Complement C3c alpha chain fragment 1;Complement C3dg fragment;Complement C3g fragment;Complement C3d fragment;Complement C3f fragment;Complement C3c alpha chain fragment 2	C3
Isocitrate dehydrogenase [NADP] cytoplasmic	IDH1
Fibronectin;Anastellin;Ugl-Y1;Ugl-Y2;Ugl-Y3	FN1
Adenosylhomocysteinase	AHCY
Ig kappa chain V-I region HK102	IGKV1-5
Desmoglein-1	DSG1
Protein disulfide-isomerase A3	PDIA3
NA	IGLV2-14
Triosephosphate isomerase	TPI1
Ig kappa chain V-II region RPMI 6410	IGKV2-30;IGKV2D-30
Caspase-14;Caspase-14 subunit p17, mature form;Caspase-14 subunit p10, mature form;Caspase-14 subunit p20, intermediate form;Caspase-14 subunit p8, intermediate form	CASP14
Endoplasmin	HSP90B1
14-3-3 protein zeta/delta	YWHAZ
Carbonic anhydrase 3	CA3
Protein disulfide-isomerase	P4HB
NA	IGHM
60 kDa heat shock protein, mitochondrial	HSPD1
Keratin, type II cytoskeletal 5	KRT5
Phosphatidylinositol-glycan-specific phospholipase D	GPLD1
Fibronectin;Anastellin;Ugl-Y1;Ugl-Y2;Ugl-Y3	FN1
NA	IGKC;IGKV1-8
NA	IGLV3-10

NA	IGKV2D-29
Keratin, type I cuticular Ha1	KRT31
Ig gamma-4 chain C region	IGHG4
Catalase	CAT
Aldehyde dehydrogenase, mitochondrial	ALDH2
Inter-alpha-trypsin inhibitor heavy chain H2	ITIH2
Histone H4	HIST1H4A
NA	IGKV1-27
Glyceraldehyde-3-phosphate dehydrogenase	GAPDH
Ig kappa chain V-I region BAN	NA
Ig kappa chain V-III region GOL	NA
Heparin cofactor 2	SERPIND1
Zinc-alpha-2-glycoprotein	AZGP1
Serotransferrin	TF
Plastin-2	LCP1
Dopamine beta-hydroxylase;Soluble dopamine beta-hydroxylase	DBH
Ornithine carbamoyltransferase, mitochondrial	OTC
Attractin	ATRN
Prolactin-inducible protein	PIP
Coagulation factor IX;Coagulation factor IXa light chain;Coagulation factor IXa heavy chain	F9
	NA
Keratin, type II cytoskeletal 78	KRT78
NA	IGLV9-49
Transmembrane protein 198	TMEM198
Complement factor H-related protein 2	CFHR2
Carboxypeptidase N catalytic chain	CPN1
NA	TF
Ig kappa chain V-III region HAH	IGKV3-20

NA	NA
Complement C1s subcomponent;Complement C1s subcomponent heavy chain;Complement C1s subcomponent light chain	C1S
Plasminogen;Plasmin heavy chain A;Activation peptide;Angiostatin;Plasmin heavy chain A, short form;Plasmin light chain B	PLG
NA	IGKV1-39;IGKV1D-39
Retinol-binding protein 4;Plasma retinol-binding protein(1-182);Plasma retinol-binding protein(1-181);Plasma retinol-binding protein(1-179);Plasma retinol-binding protein(1-176)	RBP4
Pyruvate carboxylase, mitochondrial	PC
Coagulation factor XIII A chain	F13A1
S-adenosylmethionine synthase isoform type-1	MAT1A
Apolipoprotein C-II;Proapolipoprotein C-II	APOC4-APOC2;APOC2
Arginase-1	ARG1
Lumican	LUM
Keratin, type I cytoskeletal 17	KRT17
L-selectin	SELL
Cofilin-1	CFL1
Keratinocyte proline-rich protein	KPRP
Ig lambda chain V-II region BUR	NA
NA	NA
Ig heavy chain V-III region GAR	NA
NA	IGLV3-19
Hemopexin	HPX
Ig lambda chain V-III region LOI	NA
Coagulation factor X;Factor X light chain;Factor X heavy chain;Activated factor Xa heavy chain	F10
Apolipoprotein A-IV	APOA4
Period circadian protein homolog 2	PER2

Galectin-3-binding protein	LGALS3BP
Ig kappa chain V-III region IARC/BL41	NA
Apolipoprotein C-IV	APOC4
Ig kappa chain V-I region Mev	NA
Filaggrin	FLG
Talin-1	TLN1
Ig lambda chain V-I region VOR	NA
Serum paraoxonase/arylesterase 1	PON1
NA	IGHV3-15
NA	IGHG1
Apolipoprotein C-III	APOC3
Junction plakoglobin	JUP
NA	IGKV1-16
Phospholipid transfer protein	PLTP
Desmocollin-1	DSC1
25-hydroxycholesterol 7-alpha-hydroxylase	CYP7B1
Hepatocyte growth factor-like protein;Hepatocyte growth factor-like protein alpha chain;Hepatocyte growth factor-like protein beta chain	MST1
NA	NA
Selenoprotein P	SEPP1
Protein Z-dependent protease inhibitor	SERPINA10
Suprabasin	SBSN
Metalloendopeptidase;Meprin A subunit alpha	MEP1A
NA	NA
CD44 antigen	CD44
Insulin-like growth factor-binding protein 3	IGFBP3
Keratin, type II cytoskeletal 4	KRT4
Adiponectin	ADIPOQ

Polymeric immunoglobulin receptor;Secretory component	PIGR
Ig kappa chain V-IV region STH	NA
Filaggrin-2	FLG2
Complement C1q subcomponent subunit C	C1QC
Complement C4-A;Complement C4 beta chain;Complement C4-A alpha chain;C4a anaphylatoxin;C4b-A;C4d-A;Complement C4 gamma chain	C4A
NA	IGLV3-21
Ig gamma-2 chain C region	IGHG2
NA	IGHG1
Vitamin K-dependent protein C;Vitamin K-dependent protein C light chain;Vitamin K-dependent protein C heavy chain;Activation peptide	PROC
Complement C1r subcomponent-like protein	C1RL
Biotinidase	BTD
Kininogen-1;Kininogen-1 heavy chain;T-kinin;Bradykinin;Lysyl-bradykinin;Kininogen-1 light chain;Low molecular weight growth-promoting factor	KNG1
Apolipoprotein(a)	LPA
Ig heavy chain V-III region JON	NA
Complement C1r subcomponent;Complement C1r subcomponent heavy chain;Complement C1r subcomponent light chain	C1R
Ig lambda-7 chain C region	IGLC7
NA	IGHV4-4
Complement component C9;Complement component C9a;Complement component C9b	C9
Ig kappa chain V-II region TEW;Ig kappa chain V-II region GM607	IGKV2D-28
Insulin-like growth factor-binding protein complex acid labile subunit	IGFALS
Prothrombin;Activation peptide fragment 1;Activation peptide fragment 2;Thrombin light chain;Thrombin heavy chain	F2
Ig lambda chain V-II region NIG-84	NA
Apolipoprotein L1	APOL1

NA	IGHV3-74;IGHV3-66;IGHV3OR16-13
Apolipoprotein M	APOM
Complement C5;Complement C5 beta chain;Complement C5 alpha chain;C5a anaphylatoxin;Complement C5 alpha chain	C5
Kallistatin	SERPINA4
NA	IGLV1-40
Desmoplakin	DSP
Haptoglobin-related protein	HPR
Keratin, type II cuticular Hb6	KRT86
Keratin, type I cytoskeletal 16	KRT16
Complement C2;Complement C2b fragment;Complement C2a fragment	C2
NA	IGKC
NA	IGHV4-34
Plasma serine protease inhibitor	SERPINA5
NA	IGHV3-49
Hornerin	HRNR
NA	NA
Ig kappa chain V-IV region Len	NA
Alpha-2-macroglobulin	A2M
NA	IGLV6-57
Mannan-binding lectin serine protease 1;Mannan-binding lectin serine protease 1 heavy chain;Mannan-binding lectin serine protease 1 light chain	MASP1
NA	IGHD
NA	IGLV3-25
Ig kappa chain V-I region CAR	NA
Ig kappa chain V-III region SIE	NA

EGF-containing fibulin-like extracellular matrix protein 1	EFEMP1
Ig lambda chain V-III region SH	NA
Coagulation factor XIII B chain	F13B
Ig heavy chain V-III region TRO	NA
Inter-alpha-trypsin inhibitor heavy chain H3	ITIH3
Ig kappa chain C region	IGKC
Apolipoprotein F	APOF
NA	IGHV5-51
NA	NA
Plasma kallikrein;Plasma kallikrein heavy chain;Plasma kallikrein light chain	KLKB1
Serum albumin	ALB
NA	IGHG3
Afamin	AFM
Ficolin-3	FCN3
Betaine--homocysteine S-methyltransferase 1	BHMT

NA= not available, ie unknown (n=46)
